# Cross-correlations between currents and tunnel magnetoresistance in interacting double quantum dot-Majorana wire system

**DOI:** 10.1038/s41598-024-58344-9

**Published:** 2024-04-03

**Authors:** Kacper Wrześniewski, Ireneusz Weymann

**Affiliations:** Faculty of Physics, Institute of Spintronics and Quantum Information, Adam Mickiewicz University, Uniwersytetu Poznańskiego 2, 61-614 Poznan, Poland

**Keywords:** Quantum dots, Nanowires, Electronic devices

## Abstract

We theoretically investigate the spin and charge transport properties of a double quantum dot coupled to distinct edges of the nanowire hosting Majorana zero-energy modes. The focus is on the analysis of the currents flowing through the left and right junctions and their cross-correlations. We show that the system reveals very different transport properties depending on the detuning protocol of the quantum dot energy levels. For the symmetric detuning, the current dependencies reveal only two maxima associated with resonant tunneling, and currents in the left and right arms of the system reveal weak positive cross-correlations. On the other hand, for antisymmetric detuning, the flow of electrons into drains is maximized and strongly correlated in one bias voltage direction, while for the opposite bias direction a spin blockade is predicted. Furthermore, we observe a suppression of the current cross-correlations at a highly symmetric detuning point, indicating the involvement of the Majorana zero-energy modes in the transport processes. To gain insight into the role of the spin polarization of the Majorana edge states, we analyze the spin-dependent transport characteristics by considering the relationship between the spin canting angle, which describes the coupling of the Majorana modes to the spin of the quantum dots, and the magnetic configurations of the ferromagnetic drains. Moreover, we examine the non-local zero bias anomaly in the differential conductance, detailed analysis of which revealed a specific operational mode of the device that can facilitate the identification of the Majorana presence in the quantum dot-Majorana wire system. Finally, we also consider the transport properties in different magnetic configurations of the system and discuss the behavior of the associated tunnel magnetoresistance.

## Introduction

The Majorana zero-energy modes (MZM) are quasiparticles that can emerge at the ends of a topological superconducting nanowire^1,2^. Such a nanowire provides an implementation of the Kitaev chain^3^ and, thus, its edge modes reveal a non-trivial topology^4,5^. Recently, there have been many theoretical and experimental efforts^6–11^ to deepen the knowledge and provide a better understanding of the behavior of Majorana quasiparticles in solid state systems, stimulated by promising perspectives for future applications in topological quantum computation^4,12,13^. Moreover, in-depth studies of systems hosting Majorana modes may also provide interesting results for the manipulation of topological states, for spintronic applications or for identifying hallmarks of Majorana states and other correlations in these hybrid devices. On the other hand, by being their own antiparticles and by possessing high robustness against external perturbations, Majorana zero-energy modes are also very fascinating from the perspective of fundamental physics^14^. Therefore, providing further insight into the behavior of these exotic quasiparticles, predicted by Ettore Majorana almost a century ago^15^, is of vital importance.

A very interesting and advantageous aspect of research in this field comprises the studies of the influence of MZMs on the transport properties of low-dimensional hybrid systems, such as quantum dots connected to topological superconducting nanowires^16–18^. It has been shown that Majorana modes can leak into attached quantum dots, giving rise to fractional values of the conductance through the system^19–23^. In fact, combining quantum dots with Majorana wires, allows one to explore the interplay between various phenomena exhibited by quantum dot systems^24–35^ with topologically-protected states. Interestingly, the chains of coupled quantum dots proximitized by an *s*-wave superconductor have been proposed for the implementation of the Kitaev chain in a tunable bottom-up fashion^36–38^. This has been very recently demonstrated experimentally in a device comprising double and triple quantum dots^39–41^. Moreover, it turns out that attaching quantum dots to the ends of Majorana chain can provide further means to control and explore the properties of the Majorana bound states^42,43^. From this perspective, it is crucial to provide further insight into the transport behavior of hybrid Majorana-quantum dot systems.

**Figure 1:**
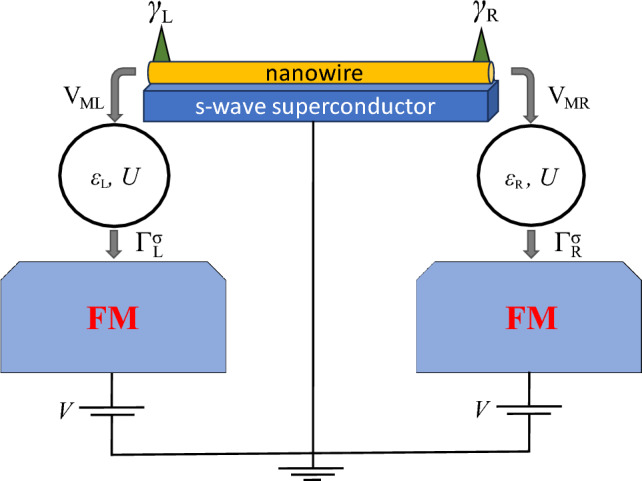
Schematic of the considered Majorana-double quantum dot system. It consists of two quantum dots coupled to opposite Majorana edge states of the common nanowire (with matrix elements $V_{\text{ML}}$ and $V_{\text{MR}}$), described by $\gamma _{\text{L}}$ and $\gamma _{\text{R}}$, and weakly coupled to ferromagnetic leads with couplings $\Gamma _{\text{L}}$ and $\Gamma _{\text{R}}$, for the left and right side, respectively. The nanowire is assumed to be grounded, while the bias voltage *V* is applied to the ferromagnetic electrodes. The quantum dots’ energy levels are denoted by $\varepsilon _{\text{L}}$ and $\varepsilon _{\text{R}}$, while *U* stands for on-dot Coulomb correlations.

In this work we therefore focus on the analysis of the non-equilibrium transport properties of a double quantum dot-Majorana wire device, which is schematically displayed in Fig. 1. In particular, we study the current, differential conductance and cross-correlations between transport processes through the opposite edges of the system. Recent theoretical and experimental studies have shown that the current fluctuations and cross-correlations can reveal additional signatures of the presence of MZM in the system^44–53^. It is also important to note that similar models as studied in this paper have already been considered, however, mostly assuming noninteracting quantum dots, indicating that the analysis of fluctuations and, specifically, current cross-correlations provides extra insight into the Majorana physics^16,44–46^. Here, we extend these studies by taking into account interaction effects in the two quantum dots connected through Majorana wire and additionally attached to ferromagnetic leads^54–56^, with an endeavor to understand the role of the Coulomb repulsion in hybridization of MZM and quantum dot states^57^ and its impact on transport characteristics. Moreover, we also examine the magnetoresistive properties of the system by exploiting ferromagnetic drains and resolving transport for wide range of the canting angle describing the spin-polarization of the Majorana edge states^58^. Interestingly, exploring the spin-selective transport characteristics have proved to be very useful in the context of Majorana polarization^53,59–63^ or Majorana spintronics^64^. Furthermore, ferromagnetic contacts turned out to be crucial for detecting entanglement in a similar system with normal superconductor instead of a topological one^65–67^.

All this indicates that it is important to provide further understanding of nonequilibrium transport through hybrid Majorana-double quantum dot systems, especially when on-dot correlations are taken into account and the tunneling processes are spin-dependent. Our work addresses this problem by using the real-time diagrammatic technique^68–71^. In particular, we identify regions of enhanced current cross-correlations, depending on particular detuning of quantum dot energy levels and value of the spin canting angle. While for antisymmetric detuning, there is a general suppression of cross-correlations, for symmetric detuning we observe a suppression of positive correlations and development of negative ones, which happens at low bias voltages as the spin canting angle is increased. Moreover, we also examine the behavior of the zero-bias anomaly due to the presence of Majorana zero-energy modes and show that, depending on the quantum dot detuning and the system’s magnetic configuration, it can exhibit either a strong dependence or this dependence is very weak. Finally, we predict an enhanced magnetoresistance of the system, which changes sign as the spin canting angle is varied. Our work thus reveals further signatures of Majorana quasiparticles in the spin-selective transport characteristics, especially in the current cross-correlations and the tunnel magnetoresistance of the system.

## Results

We consider a double quantum dot system, in which the dots are coupled through the edges of the superconducting topological nanowire hosting Majorana zero-energy modes. Each quantum dot is further attached to its own ferromagnetic lead. The bias voltage is applied between the metallic leads and the nanowire, which is assumed to be grounded. The schematic of the system is shown in Fig. 1.

### Model

The double quantum dot coupled to topological superconducting wire hosting Majorana zero-energy modes at its ends can be modelled by the following low-energy effective Hamiltonian

1 $$\begin{aligned} H=H_{\mathrm {DQD-M}}+H_{\text{Leads}}+H_{\text{Tun}}. \end{aligned}$$

Here, the first term describes the double quantum dot-Majorana subsystem^72–74^

$$\begin{aligned} H_{\mathrm{DQD-M}}=&\sum _{i=\text{L,R}}\left( \varepsilon _i n_i + U d^\dagger _{\uparrow i} d_{\uparrow i} d^\dagger _{\downarrow i} d_{\downarrow i}\right)&\\&+ \sqrt{2}V_{\text{ML}}\left[ \cos ({\theta /2})\left( d^\dagger _{\uparrow \text{L}} \gamma _{\text{L}} + \gamma _{\text{L}} d_{\uparrow \text{L}}\right) + \sin ({\theta /2})\left( d^\dagger _{\downarrow \text{L}} \gamma _{\text{L}} + \gamma _{\text{L}} d_{\downarrow \text{L}} \right) \right]&\\&-\sqrt{2}V_{\text{MR}}\left[ \cos ({\theta /2})\left( d^\dagger _{\uparrow \text{R}} \gamma _{\text{R}} + \gamma _{\text{R}} d_{\uparrow \text{R}}\right) + \sin ({\theta /2})\left( d^\dagger _{\downarrow \text{R}} \gamma _{\text{R}} + \gamma _{\text{R}} d_{\downarrow \text{R}} \right) \right]&\\&+ 2i\varepsilon _{\text{M}} \gamma _{\text{L}} \gamma _{\text{R}}, \end{aligned}$$

where the level occupation is expressed as $n_i=n_{\uparrow i} + n_{\downarrow i}=d^\dagger _{\uparrow i} d_{\uparrow i} + d^\dagger _{\downarrow i} d_{\downarrow i}$, with $d^\dagger _{\sigma i}$ ($d_{\sigma i}$) being the fermionic creation (annihilation) operator of an electron with spin $\sigma$ in the $i=\mathrm{L/R}$ quantum dot. The on-site Coulomb interaction is denoted by *U* (assumed to be the same for each dot), while the energy of the electron on the quantum dot is denoted by $\varepsilon _i$. The operators $\gamma _{\text{L}}$ and $\gamma _{\text{R}}$ correspond to the Majorana quasiparticles at the edges of the wire. These operators satisfy $\gamma _i = \gamma _i^\dag$. The third term of $H_\mathrm{DQD-M}$ describes the coupling between each quantum dot and topological superconductor, where $V_{\text{M}i}$ is the corresponding tunnel matrix element modulated by the function of the spin canting angle $\theta$^58^. In the following, we assume symmetric coupling values $V_{\text{ML}}=|V_{\text{MR}}|=V_\text{M}$. Finally, the last term describes the overlap amplitude $\varepsilon _{\text{M}}$ between the two Majorana fermions at the opposite ends of the wire.

The quantum dots are coupled to external ferromagnetic leads described by reservoirs of non-interacting quasiparticles

2 $$\begin{aligned} H_{\text{Leads}}=\sum _{i=\text{L,R}}\sum _{{\textbf {k}}\sigma }\varepsilon _{i{\textbf {k}}\sigma } c^\dagger _{i{\textbf {k}}\sigma } c_{i{\textbf {k}}\sigma }, \end{aligned}$$

where $c^\dagger _{i{\textbf {k}}\sigma }$($c_{i{\textbf {k}}\sigma }$) is the creation (annihilation) operator of an electron with momentum ${\textbf {k}}$, spin $\sigma$ and energy $\varepsilon _{i{\textbf {k}}\sigma }$ in the left ($i=\text{L}$) and right ($i=\text{R}$) lead. Finally, the last term of the system Hamiltonian specifies the tunnel coupling between the quantum dots and the leads, and is given by

3 $$\begin{aligned} H_{\text{Tun}}=\sum _{i=\text{L,R}}\sum _{{\textbf {k}} \sigma } V_{i{\textbf {k}}\sigma } \left( c^\dagger _{i{\textbf {k}}\sigma }d_{i \sigma } + \mathrm{H.c.}\right) , \end{aligned}$$

with $V_{i{\textbf {k}}\sigma }$ being the corresponding tunnel matrix elements, henceforth assumed to be spin and momentum independent, $V_{i{\textbf {k}}\sigma }\equiv V_i$. The dot-lead couplings are expressed as, $\Gamma ^\sigma _i = 2 \pi \rho _{i \sigma } |V_{i}|^2$, with $\rho _{i \sigma }$ being the spin-dependent density of states of ferromagnetic electrode *i*. The spin-dependent coupling strengths can be conveniently expressed by introducing spin polarization of magnetic leads, given by $p_i=(\rho _{i+}-\rho _{i-})/(\rho _{i+}+\rho _{i-})$, as $\Gamma ^{\pm }_i=\Gamma _i(1\pm p_i)$, where $\sigma =\pm$ denotes the majority/minority-spin subband. In the following, we assume $\Gamma _{i}=(\Gamma ^+_i+\Gamma ^-_i)/2$, $\Gamma _{\text{L}}=\Gamma _\text{R}\equiv \Gamma$, and equal spin polarization of the ferromagnetic electrodes $p_{\text{L}}=p_{\text{R}}\equiv p$. In calculations we set $p=0.5$. Furthermore, it is assumed that the system is biased by applying equal potential to both leads $\mu _{\text{L}}=\mu _{\text{R}}=eV$, while the nanowire remains grounded, see Fig. 1.

To study the nonequilibrium currents and their correlations we use the real-time diagrammatic technique^68–71^, which is described in greater detail in the Methods section. We calculate the total current as a sum of the currents flowing through the left and right ferromagnetic junctions

4 $$\begin{aligned} I=I_{\text{L}}+I_{\text{R}} \end{aligned}$$

and find the differential conductance from

5 $$\begin{aligned} G=\frac{dI}{dV}. \end{aligned}$$

On the other hand, the zero-frequency cross-correlations between the left and right currents are obtained from

6 $$\begin{aligned} S_{\text{LR}}=\int _{- \infty }^{\infty }dt\langle \delta I_{\text{L}}(t) \delta I_{\text{R}}(0) + \delta I_{\text{R}}(0) \delta I_{\text{L}}(t) \rangle , \end{aligned}$$

with $\delta I_{i}(t)={\hat{I}}_i(t)-\langle {\hat{I}}_i \rangle$ and ${\hat{I}}_{i}$ being the current operator describing tunneling between the dot and lead *i*.

Due to the spin polarization of Majorana modes in topological nanowire, the current flow is strongly dependent on the mutual alignment of magnetic moments of ferromagnetic leads. To quantify this effect, we also evaluate the tunnel mangetoresistance (TMR), which can be defined in the following way^75^

7 $$\begin{aligned} \text{TMR}=\frac{I^{\text{P}}-I^{\text{AP}}}{I^{\text{AP}}}, \end{aligned}$$

where $I^{\mathrm{P(AP)}}$ is the current flowing through both junctions when magnetic moments of the leads are aligned in parallel (antiparallel). Here, we note that due to the Majorana-dot coupling dependent on the spin canting angle, the parallel configuration (P) is assumed to be aligned along the *z*-axis of the quantization axis pointing the positive direction, while the antiparallel configuration (AP) has the left moment aligned in the opposite direction. More detailed expressions for the introduced quantities, formulated withing the real-time diagrammatic framework, are presented in the further section which describes the method.

In order to determine the transport properties, one has to find the eigenenergies and eigenvalues of the Majorana wire-double quantum dot Hamiltonian decoupled from the ferromagnetic leads, $H_{\mathrm{DQD-M}}|\chi \rangle =\varepsilon _{\chi }|\chi \rangle$. The local states of this Hamiltonian can be denoted as, $|\chi _\text{L},\chi _{\text{R}},\chi _{\text{M}}\rangle$, where $\chi _{\mathrm{L/R}} = \{0,\uparrow ,\downarrow , d \}$, standing for empty, singly-occupied with spin-up/spin-down electron and doubly occupied level of quantum dot *i*, and $\chi _{\text{M}} = \{0,1\}$. In general, due to large Hilbert space, for arbitrary model parameters, the above eigensystem needs to be solved numerically, as the eigenstates are complex functions of parameters. Nevertheless, to gain some insight into the behavior of the system, it is still possible to obtain the eigenenergies for $\varepsilon _{\text{M}}=0$. The relevant eigenenergies $\varepsilon _\chi \equiv \varepsilon _l^{\alpha , \beta }$, with $\alpha ,\beta =\pm$ and $l=1, 2, 3, 4$, are presented in Table 1. It turns out that the eigenspectrum is independent of the spin canting angle $\theta$. However, one needs to keep in mind that the magnetotransport properties of the system are sensitive to variation of this parameter, as the weights of spin components of the relevant states depend on $\theta$. We note that in the case of $\varepsilon _{\text{M}}=0$ all eigenstates with given eigenenergies $\varepsilon _\chi$ are two-fold degenerate.

**Table 1:** The eigenenergies $\varepsilon _\chi \equiv \varepsilon _l^{\alpha , \beta }$ of the Hamiltonian $H_{\mathrm{DQD-M}}$. Each eigenenergy is two-fold degenerate and the parameters $\alpha ,\beta =\pm$.

Eigenenergy $\varepsilon _l^{\alpha , \beta }$	$\Delta _1=$	$\Delta _2=$
$\varepsilon _{1}^{\alpha ,\beta }=\frac{1}{2}\left( \varepsilon _{\text{L}}+\varepsilon _{\text{R}} +\alpha \sqrt{\Delta _1+\beta \Delta _2}\right)$	$\varepsilon _{\text{L}}^2+\varepsilon _{\text{R}}^2+8V_{\text{M}}^2$	$2\sqrt{\varepsilon _{\text{L}}^2+4V_{\text{M}}^2}\sqrt{\varepsilon _{\text{R}}^2+4V_{\text{M}}^2}$
$\varepsilon _{2}^{\alpha ,\beta }=\frac{1}{2}\left( 3\varepsilon _{\text{L}}+\varepsilon _{\text{R}}+U+\alpha \sqrt{\Delta _1+\beta \Delta _2}\right)$	$\varepsilon _{\text{R}}^2+(\varepsilon _{\text{L}}+U)^2+8V_{\text{M}}^2$	$2\sqrt{(\varepsilon _{\text{L}}+U)^2+4V_{\text{M}}^2}\sqrt{\varepsilon _{\text{R}}^2+4V_{\text{M}}^2}$
$\varepsilon _{3}^{\alpha ,\beta }=\frac{1}{2}\left( \varepsilon _{\text{L}}+3\varepsilon _{\text{R}}+U+\alpha \sqrt{\Delta _1+\beta \Delta _2}\right)$	$\varepsilon _{\text{L}}^2+(\varepsilon _{\text{R}}+U)^2+8V_{\text{M}}^2$	$2\sqrt{(\varepsilon _{\text{R}}+U)^2+4V_{\text{M}}^2}\sqrt{\varepsilon _{\text{L}}^2+4V_{\text{M}}^2}$
$\varepsilon _{4}^{\alpha ,\beta }=\frac{1}{2}\left( 3\varepsilon _{\text{L}}+3\varepsilon _{\text{R}}+2U+\alpha \sqrt{\Delta _1+\beta \Delta _2}\right)$	$(\varepsilon _{\text{L}}+U)^2+(\varepsilon _{\text{R}}+U)^2+8V_{\text{M}}^2$	$2\sqrt{(\varepsilon _{\text{L}}+U)^2+4V_{\text{M}}^2}\sqrt{(\varepsilon _{\text{R}}+U)^2+4V_{\text{M}}^2}$

In the following, we present and discuss the numerical results on the current, differential conductance, cross-correlations and TMR as a function of the bias voltage and position of the quantum dot energy levels. Since this position can be changed by the gate voltage, the figures will effectively present the bias and gate voltage scans of the system transport properties. Moreover, we will distinguish two different schemes for tuning the positions of the quantum dots energy levels, depending on whether $\varepsilon _\text{L}=\varepsilon _{\text{R}}$ or $\varepsilon _{\text{L}}=-\varepsilon _\text{R}$. The first scheme will be referred to as symmetric gate detuning, while the second one as antisymmetric gate detuning. Such gating protocols will allow us to analyze the transport properties in the two antipodal detuning configurations, where the quantitative and qualitative differences are maximally embossed. We also note that similar considerations have been performed for quantum dots coupled to conventional superconductors, which implement nanoscale Cooper pair splitters^76,77^.

### The case of symmetric gate detuning

**Figure 2:**
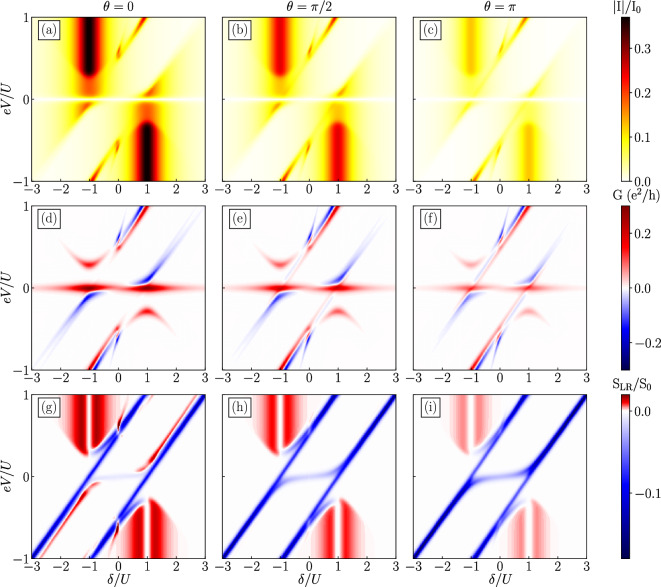
The absolute value of the current [(**a**)–(**c**)], the differential conductance [(**d**)–(**f**)] and the current-current cross-correlations [(**g**)–(**h**)] as a function of symmetric detuning $\delta$ and applied voltage *eV* for different values of spin canting angle $\theta$ indicated above the top panels. The parameters are: $U\equiv 1$ used as the energy unit, $V_{\text{M}}=0.1$, $\Gamma =0.01$, $p=0.5$, $T=0.02$ and $\varepsilon _{\text{M}}=0$. The current is normalized by $I_0=e\Gamma /\hbar$, while cross-correlations by $S_0=e^2\Gamma /\hbar$.

In this section we assume that applied scheme of gate detuning is symmetric, i.e. $\varepsilon \equiv \varepsilon _\text{L}=\varepsilon _{\text{R}}$, while the parameter $\delta =2\varepsilon +U$ describes detuning from the particle-hole symmetric point of orbital energy level, $\varepsilon =-U/2$. Additionally, the magnetic moments of ferromagnetic electrodes are assumed to form the parallel alignment. Figure 2 displays the dependencies of the absolute value of the current (a–c), the differential conductance (d–f) and the current cross-correlations (g–i) as a function of applied bias voltage *V* and level detuning $\delta$ for selected values of $\theta$.

The results are presented in three columns for different values of the spin canting angle $\theta$. We start the analysis of charge transport through the considered system by examining the obtained dependencies for $\theta =0$. For $eV=0$, the current does not flow through the system, however, the differential conductance clearly reveals a strong zero-bias peak associated with the Majorana modes leaking into the quantum dots. We note that the basic dependencies of the zero-bias anomaly on various model parameters have recently been studied in a hybrid Majorana system with one quantum dot coupled to ferromagnetic leads^53^. However, in the case of the double quantum dot-Majorana device considered here, we have the opportunity to study the behavior of a non-local zero-bias anomaly revealed in magnetotransport properties, which will be comprehensively discussed later on.

The general current dependence is antisymmetric with respect to the reversal of the bias voltage and detuning, $I(\delta , eV)=-I(-\delta , -eV)$ and assuming $\theta =0$. The main features revealed in the current-voltage spectra are the two maxima of the absolute current present for $\delta /U=\pm 1$, which are due to resonant position of quantum dot and Majorana levels. Let us focus on the case of $\delta /U=1$ detuning, while the opposite case of $\delta /U=-1$ can be explained by similar reasoning, but considering different eigenstates. The current in the low bias voltage region is then facilitated by the two-fold degenerate ground state of energy $\varepsilon _1^{-,+}$, described by the many-body states which can be approximately expressed as $|\chi \rangle \approx \frac{1}{2}|\uparrow , 0, 1\rangle + \frac{1}{2}|0, \uparrow , 1\rangle + \frac{1}{\sqrt{2}}|0, 0, 0\rangle$ and $|\chi '\rangle \approx \frac{1}{2}|\uparrow , 0, 0\rangle + \frac{1}{2}|0, \uparrow , 0\rangle + \frac{1}{\sqrt{2}}|0, 0, 1\rangle$. When the applied bias voltage is increased above $|eV/U|\gtrsim 0.2$, another excited state enters the transport window, which is associated with appearance of the consecutive step in the current-voltage characteristics. This threshold voltage is exactly equal to the difference between $\varepsilon _1^{-,+}$ and $\varepsilon _1^{-,-}$, which is equal to $2V_{\text{M}}$. For reversed bias voltage, however, the current remains blocked by a quantum dot spin configuration opposite to the Majorana state. More specifically, the double quantum dot-Majorana system is then trapped in states ${|\chi \rangle \approx \frac{1}{\sqrt{2}}\left( |\downarrow , 0, 0\rangle + |0, \downarrow , 0\rangle \right) }$ and ${|\chi '\rangle \approx \frac{1}{\sqrt{2}}\left( |\downarrow , 0, 1\rangle + |0, \downarrow , 1\rangle \right) }$ of approximately equal probability.

Further valuable transport properties are exposed in the current cross-correlations (g–i). In general, for the regimes where considerable current is flowing for $\delta /U\approx \pm 1$, we predict positive but rather small values of $S_{\text{LR}}$. Importantly, for exact detuning of $\delta /U=\pm 1$, the cross-correlations are fully suppressed, $S_{\text{LR}}=0$. This suppression has been suggested as a specific feature that can distinguish presence of Majorana zero-energy modes from the Andreev bound states^45^. Such uncorrelated transport is enabled by the degeneracy of non-local zero-energy states, which is a consequence of tuning and assumed vanishing overlap between the Majorana edge states, i.e. $\varepsilon _{\text{M}}=0$.

**Figure 3:**
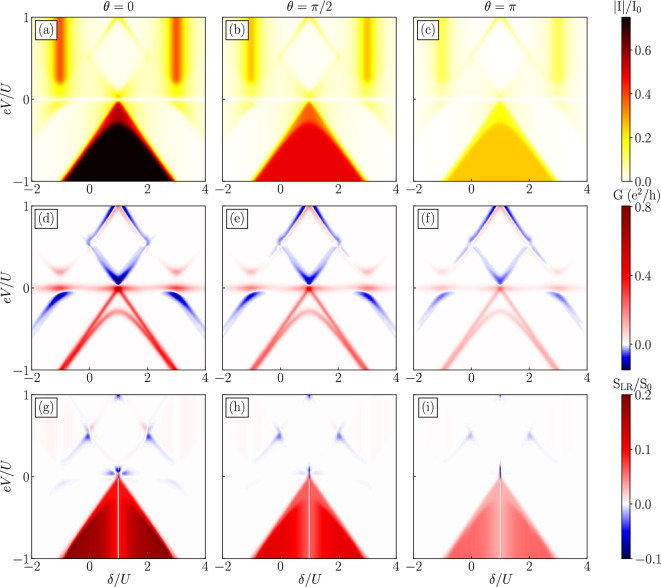
The absolute value of the current [(**a**)–(**c**)], the differential conductance [(**d**)–(**f**)] and the current-current cross-correlations [(**g**)–(**h**)] as a function of antisymmetric detuning $\delta =2\varepsilon + U$ ($\varepsilon \equiv -\varepsilon _\text{L}=\varepsilon _{\text{R}}$) and applied voltage *eV* for different values of spin canting angle $\theta$ indicated above the top panels. The other parameters are the same as as in Fig. 2.

Additionally, we observe two parallel, antidiagonal regions of strong negative cross-correlations. These regions spread in the $\delta -eV$ parameter space along the lines $eV=\delta /2+1/2$ and $eV= \delta /2-1/2$. The values of *eV* and $\delta$ associated with these lines set the energy levels of both sides of the device in such a configuration that enables tunneling processes in opposite directions in the left and right junctions.

Finally, we show that all discussed transport quantities are decreased as the spin canting angle $\theta$ is increased in the range from 0 to $\pi$. We recall that for $\theta =0$, the Majorana mode couples only to spin-up component of the electrons occupying quantum dots and this direction is aligned with parallel configuration of the ferromagnetic leads. As the angle is increased, the spin polarization of the edge state is shifted to opposite direction resulting in a decrease of the current and the remaining transport quantities. Interestingly, $\theta =\pi /2$ is the angle that gives rise to identical results to those in the case of antiparallel configuration of ferromagnetic leads, as for this angle the spin polarization is orthogonal to ferromagnetic moments. For $\theta =\pi$, the Majorana mode couples completely to opposite spin component compared to the case of $\theta =0$. In this configuration the currents and other quantities reveal the lowest values due to the bottleneck resulting from high spin polarization of electrons in direction of minority-spin band in the ferromagnetic leads.

Interestingly, only negative cross-correlations in low-bias region are noticeably amplified. This can be understood by examining thermal charge fluctuations in this regime. For $\theta =0$, fluctuating charges are highly spin-polarized due to alignment of Majorana spin polarization with magnetic moments of both the electrodes. Particles of opposite spins rarely contribute to this characteristic. However, in extreme case when $\theta =\pi$, the population of particles with spin in opposite-to-Majorana direction is increased due to single electron tunneling processes between the ferromagnetic lead and quantum dot according to the spin-dependent couplings $\Gamma ^{\pm }_i$. Given the fact that those anti-aligned electrons are not coupled to the nanowire edge state, eventually these have to tunnel back into the same electrode due to the fluctuations. Such condition significantly increases the amplitude of correlated tunneling events in opposite directions between the left and right leads, resulting in considerable negative cross-correlations.

### The case of antisymmetric gate detuning

We now focus on the analysis of transport in the case of antisymmetric gate detuning of quantum dots expressed by $\varepsilon \equiv -\varepsilon _{\text{L}}=\varepsilon _{\text{R}}$ and study the corresponding $\delta$ and *V* dependencies. We note that a similar protocol of detuning was proposed for quantum dot devices coupled to superconducting electrode in the splitter geometry^76,77^, where the energy conservation ($\varepsilon _{\text{L}}+\varepsilon _{\text{R}}=0$) gives rise to an efficient transport and maximized current flowing through the system. Figure 3 presents the dependencies of the current (a–c), the differential conductance (d–f) and the current cross-correlations (g–i) as a function of *eV* and antisymmetric level detuning $\delta$.

Qualitatively, the transport characteristics are very different when compared to the symmetric detuning case. First of all, the current mainly flows in one direction, i.e. for negative bias voltage when particles tunnel from nanowire toward the ferromagnetic drains, while in the opposite direction there is a spin blockade in a wide range of detuning and applied bias voltage that suppresses the transport. However, for $eV<0$, the absolute current is much higher than in previously considered case, and this regime is significantly wider in the parameter space forming a triangular shape, while for symmetric detuning only narrow resonances were present. The region of enhanced current for negative bias voltage is defined by $eV=-|\delta /2-1/2|$. Moreover, we note that in the case of antisymmetric detuning, the expressions for eigenenergies in Table 1 are simpler as leading terms contributing to eigenenergies cancel when $\varepsilon _{\text{L}}=-\varepsilon _\text{R}$. In this regime, in total 8 states contribute to transport processes with approximately equal probabilities, which results in considerably higher absolute value of the current compared to the case of symmetric detuning where fewer states were relevant. These states are linear combinations of local states with double quantum dots occupied by spin-up electrons. Because the spin of tunneling electrons is then aligned with the Majorana polarization, the corresponding large current is observed. On the other hand, for positive bias voltage, the current becomes suppressed due to the fact that the occupation of spin-down double dot states becomes enhanced, which is misaligned with the Majorana polarization.

Two important features associated with Majorana physics are also present in the results shown in Fig. 3. Again, we find zero-bias anomaly in the differential conductance [see Fig. 3d–f] and the current cross-correlations are suppressed in very narrow range of detuning $\delta /U=1$. Here, the valley with $S_{\text{LR}}=0$ is extremely narrow when compared with symmetric detuning case and it may be harder to explore in tunnel-spectroscopic experiments. We remind that for $\delta /U=1$ we have $\varepsilon =-\varepsilon _\text{L}=\varepsilon _{\text{R}}=0$. A small shift from $\delta /U=1$ results in splitting of orbital energy levels and putting the system away from highly degenerate point responsible for uncorrelated transport. Away from $\delta /U=1$, the cross-correlations reveal very high positive values for the whole range of parameters where current is maximized, while in this detuning protocol the negative values are generally very small for a few detuning points.

We also note that generally the manipulation of the spin canting angle $\theta$ affects the transport characteristics quantitatively in a similar way as in the case of symmetric detuning discussed earlier. As the angle $\theta$ is increased, there is a significant decrease of the differential conductance and current cross-correlations, while the qualitative features discussed in this section remain intact. However, there are also some differences, especially at low bias voltages. While in the case of symmetric detuning there was an enhancement of negative cross-correlations at low bias, no such feature is observable in the case when detuning is antisymmetric. Here, the antisymmetric level detuning of dots shifts the charge fluctuations to one side of the device effectively suppressing left-right anti-correlated processes independently of spin canting angle $\theta$.

### Zero-bias anomaly

**Figure 4:**
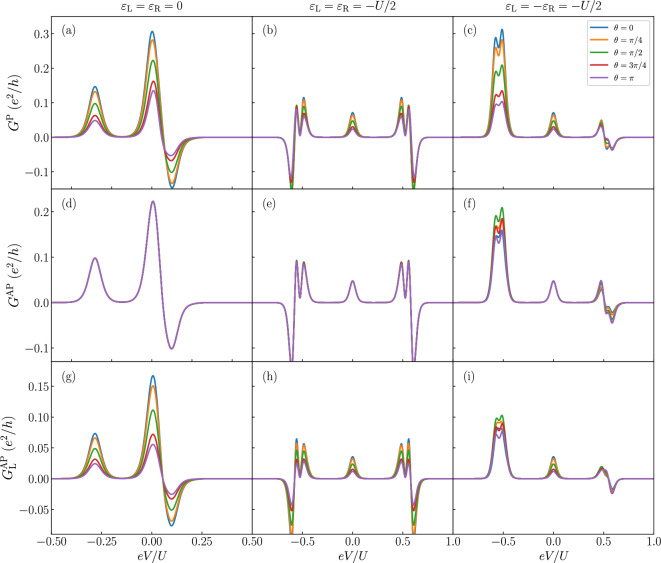
The bias voltage dependence of the differential conductance for selected values of the quantum dot level positions and different values of the spin canting angle $\theta$, as indicated. The first (second) row presents $G^{\text{P}}$ $\left( G^{\text{AP}}\right)$, while the third row shows $G^{\text{AP}}_{\text{L}}$. The other parameters are the same as in Fig. 2.

The zero-bias anomaly in the differential conductance, attributed to the presence of zero-energy modes, is regarded as a fingerprint of Majorana physics. However, in nanoscale systems, the origin of the peak may vary, and may possibly arise from the presence of Andreev bound states. It is essential to identify behaviors associated with this phenomenon that enable the experimental differentiation of the source of the conductance peak in a convenient and reliable manner.

The influence of the leads’ spin polarization, the strength of coupling to the Majorana wire, and the overlap between the two edges of the nanowire have all been previously analyzed for systems consisting of a single quantum dot^53^. Those results are quantitatively relevant to the system under consideration in this study. However, the ability to tune the spin polarization of Majorana modes and examine the conductance of both left and right junctions provides additional insight into magnetotransport and can aid in identifying Majorana physics.

Figure 4 presents the bias voltage dependence of the differential conductance in the parallel $\left( G^{\text{P}}\right)$ and antiparallel $\left( G^{\text{AP}}\right)$ magnetic configurations of the system. Additionally, we show the contribution to the conductance from the left $\left( G^{\text{AP}}_{\text{L}}\right)$ junction in antiparallel configuration, while we notice that $G^{\text{AP}}_{\text{R}}=G^{\text{AP}}-G^{\text{AP}}_\text{L}$ and, therefore, we do not show it. The presented curves are evaluated for several values of the spin canting angle $\theta$ and for three representative gate detunings, corresponding to both symmetric and antisymmetric protocols.

First of all, it is important to note that in the parallel configuration, the differential conductance strongly depends on $\theta$ for all detunings. This dependence also holds for all conductance peaks, including the zero-bias anomaly. Interestingly, in the antiparallel configuration the total conductance $G^{\text{AP}}$ is unaffected by variation of spin canting angle in symmetric detuning, see Fig. 4d,e. The change of the Majorana spin polarization results in an increased conductance in one junction, while it is counterbalanced by a proportional decrease in the opposite junction, thereby conserving the total conductance of the device. The behavior of the differential conductance associated with the current through the left junction is presented in the third row of Fig. 4. Nevertheless, the $\theta$ dependence is most interesting in the case of antisymmetric detuning, see Fig. 4f. The zero-bias peak remains unaffected by variations in the spin canting angle, while other conductance peaks arising at higher voltages are evidently modified. Magnetotransport measurements in this operating mode of the device can facilitate the identification of Majorana presence with high likelihood, as other zero-bias phenomena of different origins are not consistent with the discussed behavior, i.e. conductance of singlet Andreev bound state would be strongly affected by magnetic configuration of the drains and their polarization, especially in the antiparallel alignment.

**Figure 5:**
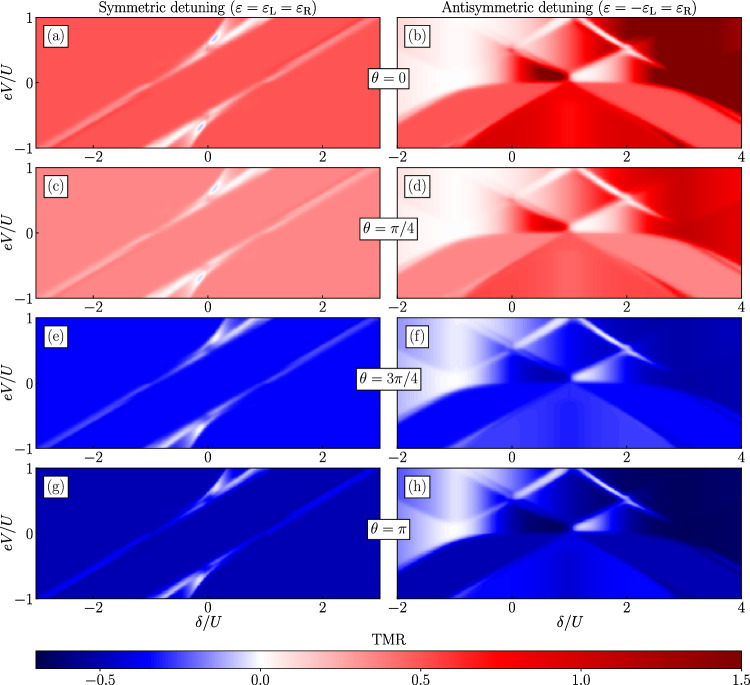
The tunnel magnetoresistance for symmetric (left column) and antisymmertric (right column) detuning as a function of $\delta$ and applied bias voltage *eV* for different values of the spin canting angle $\theta$, as indicated. The parameters are the same as in Fig. 2.

### Tunnel magnetoresistance

**Figure 6:**
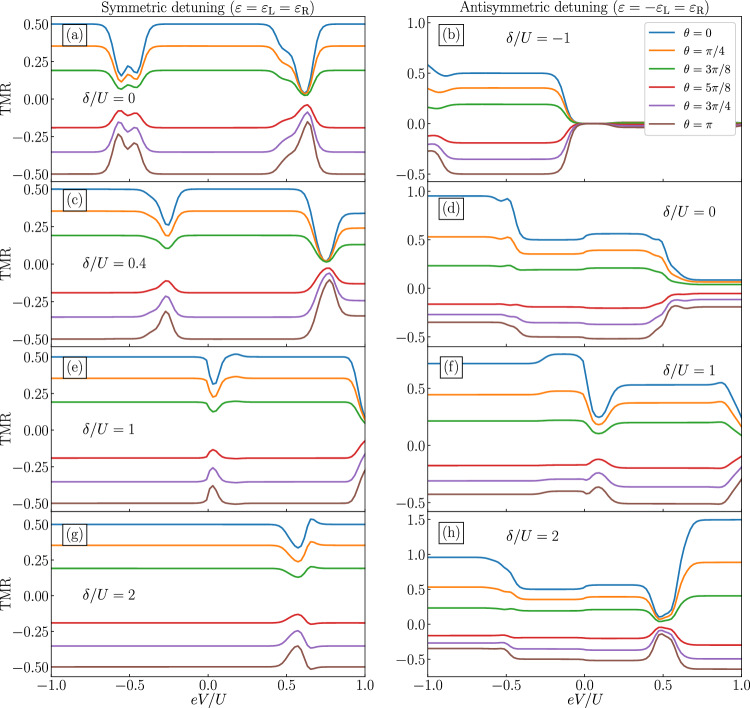
Cross-sections of the tunnel magnetoresistance for symmetric (left column) and antisymmertric (right column) detuning as a function of applied bias voltage *eV* for different values of the spin canting angle $\theta$ and $\delta$. The parameters are the same as in Fig. 2.

Finally, let us examine the behavior of the tunnel magnetoresistance for both detuning schemes and for different values of the angle $\theta$. The bias and gate voltage dependence of the TMR is shown in Fig. 5, while Fig. 6 presents the bias voltage dependence of TMR for selected values corresponding to the cross-sections of the density plots shown in Fig. 5. We recall that we find the TMR from the currents flowing in the parallel and antiparallel configurations, cf. Eq. (7). Moreover, it is necessary to stress that by considering the Majorana-dot coupling depending on the spin canting angle $\theta$, the spin symmetry is broken and one needs to be careful with identifying the possible magnetic configurations for the TMR measurements.

First of all, one can note that the general dependency of the TMR on $\delta /U$ and *eV*/*U* is antisymmetric, likewise earlier discussed quantities. Assuming that we start with the angle $\theta =0$ in the parallel configuration, for which the leads’ magnetic moments point in the same direction as the Majorana mode polarization, the TMR has generally positive and highest values for both detuning schemes, see Fig. 5a–b. For symmetric detuning, in a wide range of detuning and bias voltage, we observe a constant value of the TMR, $\text{TMR}\approx 0.5$, see also the left column of Fig. 6. On the other hand, there are two minima, where negative cross-correlations are present [cf. Fig. 2], where TMR is suppressed, which indicates that transport hardly depends on the magnetic configuration of the system. As the angle $\theta$ is increased, the TMR monotonically goes down towards negative values for $\theta >\pi /2$. For the exact value of $\theta =\pi /2$, we observe no TMR in the whole range of parameter space, as this configuration sets the Majorana state polarization in orthogonal direction with respect to the leads’ magnetic moments. As a consequence, $I^\text{P}=I^{\text{AP}}$ and, thus, $\text{TMR}=0$.

When the detuning of the dots levels is antisymmetric, the TMR reveals more compelling features. Interestingly, the magnetoresistance dependence is not mirror symmetric with respect to vertical line at $\delta /U=1$, contrary to the other quantities discussed earlier for this detuning scheme, see Fig. 3. Here, it is important to recognize that for antisymmetric detuning there are two possible non-identical antiparallel configurations, which differ in alignment of leads’ magnetic moments with respect to Majorana polarization. For the obtained results, we assumed that the right quantum dot is coupled to ferromagnetic lead with magnetic moment pointing in positive direction of z-axis, while the left quantum dot is coupled to ferromagnetic lead with magnetic moment in opposite direction. The results for opposite antiparallel configuration would reveal similar dependencies as presented here, but mirror symmetric with respect to the vertical line at $\delta /U=1$. Interestingly, for the considered case of antiparallel configuration, in the detuning regime $\delta /U\lesssim 0$, there is an area with suppressed TMR, $\text{TMR}\approx 0$. This behavior is not affected by variations of the spin canting angle $\theta$, see Fig. 6b. However, clearly, in the detuning range, for which the quantum dot coupled to the electrode with opposite direction of magnetic moment to the spin polarization of Majorana edge mode has high values of orbital level ($\delta /U \gtrsim 2$ and in consequence $\varepsilon _{\text{R}} \gtrsim 0$), the strongly polarized current is flowing through the other side (left) of the device. Such scenario generates high TMR upon switching the magnetic configuration, which approaches $\text{TMR}\rightarrow 3/2$, see Fig. 6h. This regime is very fragile to the variation of $\theta$ and as this angle is increased, we observe monotonic decrease of the TMR and, eventually, negative values for $\theta >\pi /2$. Finally, it is important to note that the highest variability of the TMR is in the transport regimes when generally small current is flowing through the system.

## Discussion

We have analyzed the charge and spin transport properties of the double quantum dot-Majorana nanowire system by means of the real-time diagrammatic technique. The calculations were performed in a perturbative manner taking into account the first-order tunneling processes in the coupling to normal leads. We determined the currents, the associated differential conductance and current cross-correlations for a wide parameter space in both the linear and nonlinear response regimes. Additionally, we considered the magnetoresistive properties of the system by analyzing the tunnel magnetoresistance associated with a relative change of the magnetic moments of the leads. Whenever possible we have also indicated the eigenstates and eigenenergies of the effective double dot-Majorana Hamiltonian responsible for the observed behavior.

In our considerations we focused on two different quantum dot level detuning scenarios. In the case of symmetric detuning, we found that significant currents flow mostly in two transport regimes, namely, where the quantum dot levels are in resonance with the respective chemical potentials of the leads, which takes place for detuning parameter $\delta /U=\pm \,1$. Interestingly, for exact values of $\delta /U=\pm \,1$, the system exhibits suppressed cross-correlations, which are however magnified in the regime around the resonance points. We demonstrated that while these cross-correlations become diminished as the spin canting angle is increased, negative current cross-correlations develop at low bias voltage for detunings between the two resonant values. On the other hand, for antisymmetric detuning, we generally observe higher values of absolute current for negatively biased system. Additionally, strong positive current cross-correlations are revealed, indicating highly correlated character of transport in this regime. For positive bias voltage, a spin blockade is formed, which is responsible for the strong current blockade. These features become suppressed with increasing the spin canting angle. Furthermore, we conducted a comprehensive analysis of the dependence of the zero-bias anomaly in the differential conductance on the spin canting angle and proposed magnetotransport measurements that can aid in identifying whether the origin of this phenomenon is associated with the Majorana physics.

Finally, the behavior of the TMR and the influence of relation between magnetic configurations of the leads and the spin canting angle were discussed. We showed that for symmetric detuning, the system reveals moderate magnetoresistive properties mainly because of highly polarized currents due to spin structure of Majorana edge states. However, for antisymmetric detuning, we found a transport regime, in which the TMR can achieve considerable values $\text{TMR}\rightarrow 3/2$. Moreover, it can be conveniently controlled in wide range of values by tuning the spin canting angle via magnetic field or quantum dots’ gate voltages. Such property gives further insight into the spin polarization of Majorana edge state.

## Methods

In order to find the current and the corresponding current cross-correlations, we use the real-time diagrammatic technique in the first-order perturbation approximation with respect to the tunnel coupling between the quantum dots and the corresponding leads^68–71^. The evaluation of diagrams with the help of diagrammatic rules allows one to find the relevant self-energies and build the ${\textbf{W}}$ matrix. The matrix elements $W_{\chi \chi '}$ quantify the transition between the eigenstates $|\chi \rangle$ and $|\chi '\rangle$ of the Hamiltonian $H_{\mathrm{DQD-M}}$. Necessary for further calculations, in a similar fashion the matrix ${\tilde{\textbf{W}}}$ is built, where one arbitrary row of ${\textbf{W}}$ is replaced with vector ($\Gamma , \Gamma ,..., \Gamma$) and a matrix $\mathbf {W^{I_i}}$ is constructed accounting for the number of electrons transferred through $i=\mathrm{L/R}$ junction^70,71^.

After solving the kinetic equation

8 $$\begin{aligned} \mathbf {Wp^{\text{st}}}=0, \end{aligned}$$

where $\mathbf {p^{\text{st}}}$ is the vector with probabilities $\text{p}^{\text{st}}_\chi$, the current through the $i=\mathrm{L/R}$ junction is calculated from^70,71^

9 $$\begin{aligned} I_i=\frac{e}{\hbar }\text{Tr}\left\{ \mathbf {W^{I_i}p^{\text{st}}}\right\} . \end{aligned}$$

Finally, the first order diagrammatic expression for current cross-correlation between left and right junctions is given by^70,71^

10 $$\begin{aligned} S_{\text{LR}}=\frac{e}{\hbar }\text{Tr}\left\{ (\mathbf {W^{I_L}P W^{I_R}+W^{I_R}P W^{I_L})p^{\text{st}}} \right\} . \end{aligned}$$

The propagator ${\textbf{P}}$ is determined from ${\tilde{\textbf{W}}}{\textbf{P}}={\textbf{p}}^{\text{st}}\mathbf {e^{\text{T}}-1}$. Here, matrix ${\tilde{\textbf{W}}}$ is build from ${\textbf{W}}$, where one row is replaced with $(\Gamma , \Gamma ,..., \Gamma )$, while $\mathbf {e^{\text{T}}}=(1, 1,..., 1)$.
